# Significant impact of forcing uncertainty in a large ensemble of climate model simulations

**DOI:** 10.1073/pnas.2016549118

**Published:** 2021-05-28

**Authors:** John C. Fyfe, Viatcheslav V. Kharin, Benjamin D. Santer, Jason N. S. Cole, Nathan P. Gillett

**Affiliations:** ^a^Canadian Centre for Climate Modelling and Analysis, Environment and Climate Change Canada, Victoria, BC V8W 2Y2, Canada;; ^b^Program for Climate Model Diagnosis and Intercomparison, Lawrence Livermore National Laboratory, Livermore, CA 94550

**Keywords:** climate change, climate model, external forcing, large ensemble

## Abstract

Forcing due to solar and volcanic variability, on the natural side, and greenhouse gas and aerosol emissions, on the anthropogenic side, are the main inputs to climate models. Reliable climate model simulations of past and future climate change depend crucially upon them. Here we analyze large ensembles of simulations using a comprehensive Earth System Model to quantify uncertainties in global climate change attributable to differences in prescribed forcings. The different forcings considered here are those used in the two most recent phases of the Coupled Model Intercomparison Project (CMIP), namely CMIP5 and CMIP6. We show significant differences in simulated global surface air temperature due to volcanic aerosol forcing in the second half of the 19th century and in the early 21st century. The latter arise from small-to-moderate eruptions incorporated in CMIP6 simulations but not in CMIP5 simulations. We also find significant differences in global surface air temperature and Arctic sea ice area due to anthropogenic aerosol forcing in the second half of the 20th century and early 21st century. These differences are as large as those obtained in different versions of an Earth System Model employing identical forcings. In simulations from 2015 to 2100, we find significant differences in the rates of projected global warming arising from CMIP5 and CMIP6 concentration pathways that differ slightly but are equivalent in terms of their nominal radiative forcing levels in 2100. Our results highlight the influence of assumptions about natural and anthropogenic aerosol loadings on carbon budgets, the likelihood of meeting Paris targets, and the equivalence of future forcing scenarios.

## Significance

Climate models are the main tool used to make projections of future climate change to inform adaptation and mitigation decisions. Confidence in these projections rests in part on the models’ ability to reproduce historical climate variations. Here we use a Earth System Model to evaluate the role of external forcing uncertainty in simulations of past and future climate change. We demonstrate that apparently small differences in anthropogenic aerosol forcing applied in the models can have a significant impact on the resulting climate simulations, as can the neglect of preindustrial and future volcanic forcings. This points to the need to reduce forcing uncertainties and better quantify their impact on the physical climate system, carbon budgets, and the Paris accord temperature targets.

Global climate models rely on forcing information to simulate past and project future climate change. Observed estimates of changes in well-mixed greenhouse gases, anthropogenic aerosols, tropospheric and stratospheric ozone, land use, solar irradiance, and volcanic aerosols are used for the past, while a range of forcing scenarios are employed for the future. On a 5- to 6-y cycle, forcing information is developed and updated under the auspices of the Coupled Model Intercomparison Project (CMIP) and made available to climate modeling groups worldwide to undertake coordinated experiments in support of scientific assessment reports produced by the Intergovernmental Panel on Climate Change (IPCC). Currently, under Phase 6 of CMIP (CMIP6) (1), model simulations relied on the latest observed forcing estimates and future forcing scenarios to support the upcoming Sixth Assessment Report of the IPCC. Under Phase 5 of CMIP (CMIP5) (2), simulations were performed using the observed forcing estimates and future forcing scenarios available over a decade ago. CMIP5 simulations supported the Fifth Assessment Report of the IPCC (3).

**Fig. 1.**
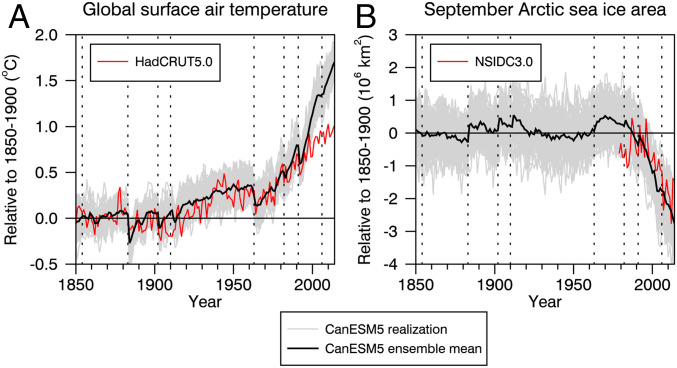
(*A* and *B*) Time series of anomalies in annual-mean and global-mean surface air temperature (*A*) and September Arctic sea ice area (*B*) in a 50-member ensemble of CanESM5 simulations employing CMIP6 forcings. The solid black curves are ensemble means and the light gray curves are the individual simulations. The red curve in *A* is based on HadCRUT5.0 observations and that in *B* is based on NSIDC3.0 observations. The vertical dotted lines indicate eruption years of the Shiveluch (1854), Krakatoa (1883), Santa Maria (1902), Novarupta (1910), Agung (1963), El Chichón (1982), Pinatubo (1991), and Tavurvur (2006) volcanoes. The reference period is 1850 to 1900. Given this preindustrial reference period and the fact that sea ice area observations are unavailable prior to 1979, we have set the observed sea ice area anomaly averaged from 1981 to 2010 to that of the model mean.

Fig. 1 shows two key metrics of global climate change from version 5 of the Canadian Earth System Model (CanESM5) (4). The model was run with the observational forcing estimates provided through CMIP6. Each of the 50 simulations is initialized from a different initial condition (*Methods and Materials*) and then yields an underlying climate “signal” in response to the applied forcing plus a unique individual realization of the “noise” of climate variability. We find simulated global warming that is consistent with in situ observations up until about 2000. After 2000, the simulated warming is greater than observed (Fig. 1*A*). Here we note that the equilibrium climate sensitivity (ECS), defined as the amount of global-mean surface warming resulting from a doubling of atmospheric ${CO}_{2}$, of this model is significantly higher than the CMIP5 mean value (5) and the CMIP6 mean value (6). It has been suggested that changes in climate feedbacks rather than forcings are the source of the higher ECS in CanESM5 (4). Indications are that the higher ECS in this model is associated with cloud and surface albedo feedbacks, with sea ice likely playing an important role in the latter effect. It is important to note that since we are reporting here on relative changes between CanESM5 simulations, we do not believe that its high ECS is a significant issue. Finally, we note that simulated Arctic sea ice loss in September is consistent with satellite observations since 1979 (Fig. 1*B*). Signals of large volcanic eruptions are evident in both observed and simulated global temperature and in observed and simulated Arctic sea ice.

How sensitive are these climate signals to reasonable but differing estimates of the applied forcing? In the following sections, we address this question, by comparing CanESM5 simulations using older CMIP5 forcings and newer CMIP6 forcings. Our focus is on three different periods: a preindustrial control period before 1850, a historical period from 1850 to 2014, and a future period from 2015 to 2100. While studies comparing simulations from multiple climate models using identical forcings in a given phase of CMIP are commonplace, studies comparing simulations from a single model using forcings from two different phases of CMIP are relatively rare (7). We note that these two approaches are complementary. The former allows for the quantification of model uncertainty and the latter for the quantification of forcing uncertainty.

## Preindustrial Control Period (before 1850)

Preindustrial control periods are periods over which climate models are run with time-invariant external forcing until they reach some quasi-equilibrium climatic state. Due to slow processes in the ocean, this typically requires hundreds to thousands of years of simulated climate. Simulations over a given historical period, such as the 1850 to 2014 period used in CMIP6, are then initiated toward the end of the control simulation. Historical integrations rely on observed estimates of the major anthropogenic and natural forcings. As we show below, assumptions made in the transition from the preindustrial to the historical period can have a pronounced impact on both historical and future simulations (8).

In CMIP6, the volcanic aerosol protocol for the preindustrial control period dictates use of a constant forcing equal to the time-averaged observed volcanic aerosol forcing over the historical period. Every modeling group participating in CMIP6 follows this protocol. The specified volcanic aerosols reflect solar radiation, cooling the atmosphere, and the land and ocean surfaces. In contrast, CMIP5 had no volcanic aerosol protocol for the preindustrial period. Some CMIP5 modeling groups used nominal background aerosol forcing while others did not. To assess the implications of this choice we conducted preindustrial control simulations with CMIP5 and CMIP6 volcanic aerosol protocols.

**Fig. 2.**
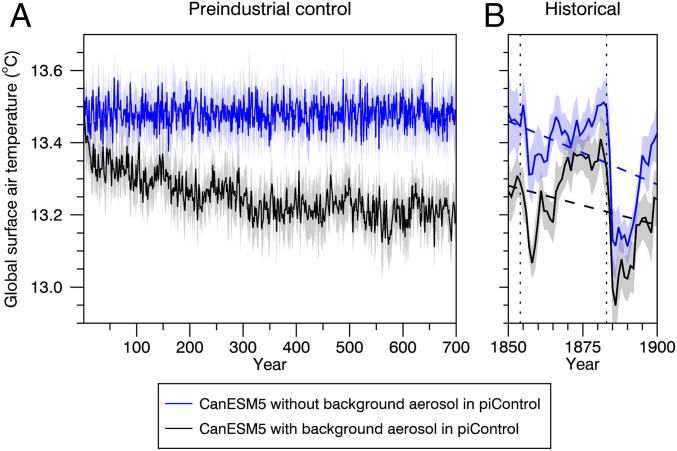
(*A* and *B*) Time series of annual-mean and global-mean surface air temperature in 10-member simulation ensembles of CanESM5 employing CMIP5 external forcings. Results are from preindustrial control simulations with time-invariant forcings (*A*) and from historical simulations with time-varying forcings (*B*) (*Materials and Methods*). The curves are ensemble means and the shadings are 95% confidence intervals on the ensemble means. The blue curves and shadings are for simulations in which the preindustrial control period employs no background volcanic aerosol forcing. The black curves and shadings are for simulations in which the preindustrial control period has background volcanic aerosol forcing equal to an estimate of the observational average over the historical period from 1850 to 2005. In *B* the dotted lines indicate eruption years of the Shiveluch (1854) and Krakatoa (1883) volcanoes, and the dashed lines are linear trend lines.

Without background volcanic aerosol forcing, the time-averaged global surface air temperature (GSAT) in the CanESM5 preindustrial control run is 13.48 °C. With background volcanic aerosol loadings in an ensemble of 10 simulations (each initialized from different points in the preindustrial simulation), the climate system cools over several hundred years. When averaged over the last 100 y of each simulation, the ensemble-mean GSAT is 13.21 °C (Fig. 2*A*). The ensemble-mean difference between these two sets of simulations is 0.27 ± 0.01 ° C (95% confidence interval).

These results have implications for the evolution of temperature over the historical period and the full 21st century. Simulations that do not incorporate volcanic aerosol forcings in their preindustrial control will necessarily undergo a cooling adjustment when they transition into the historical period that does include volcanic aerosol forcing. Fig. 2*B* shows this for the “preindustrial” period from 1850 to 1900 under time-evolving CMIP5 forcings. While the cooling adjustment in the simulations without volcanic aerosols in the preindustrial control period (blue curves in Fig. 2*B*) is obscured by the responses to the eruptions of the Shiveluch volcano in 1854 and Krakatoa in 1883, the linear temperature change in these simulations (blue dashed line in Fig. 2*B*) is larger than in the simulations with volcanic aerosols in the preindustrial control period (black dashed line in Fig. 2*B*). The difference in linear change between these two sets of simulations (∼0.074 °C) over 1850 to 1900 is comparable to the corresponding difference over the first 50 y of the preindustrial run (∼0.066 °C).

Consequently, the simulations without background volcanic aerosol forcing in the preindustrial control period warm less over the historical period. In CanESM5 under CMIP5 forcings, the warming between 1850 to 1900 and 2005 to 2014 is about 0.10±0.07 °C smaller in the set of simulations that do not include volcanic aerosol forcing in the preindustrial control period. Similar results have been obtained in the context of global sea level rise (8).

## Historical Period (1850 to 2014)

Here we compare results from the CanESM5 run with CMIP5 and CMIP6 historical forcing estimates. We consider simulations employing all forcings (ALL), greenhouse gas only (GHG), anthropogenic aerosol only (AER), and natural only external factors (volcanic and solar; NAT). To avoid the “warm start” issue discussed in the previous section, we consider only ALL and NAT simulations that were initialized under preindustrial control simulations that included background volcanic aerosol forcing. (By “background,” we mean the time-averaged volcanic aerosol loading in the historical run.) For each of these four different sets of CanESM5 simulations we have 10 historical simulations with CMIP5 forcings and 50 historical simulations with CMIP6 forcings. Before forming differences between CMIP5 and CMIP6 simulations, each time series has its average value from 1850 to 1900 removed—i.e., we are considering departures from a nominal preindustrial state. A comparison of solar forcing differences between CMIP5 and CMIP6 forcings has been published elsewhere (9). While solar forcing differences exist between CMIP5 and CMIP6, they are small and unlikely to be expressed as significant differences in near-surface climate.

The CMIP5 protocol did not dictate a specific volcanic forcing dataset. At least three different datasets were used by modeling groups. We rely on the volcanic forcing dataset (10) that was the most widely used in CMIP5 (11). In CMIP6 all of the modeling groups used one of two volcanic forcing datasets. Differences between the two CMIP6 datasets are mainly confined to the 1991 Pinatubo eruption, are relatively minor, and do not have a significant impact on simulations of global temperature (12).

**Fig. 3.**
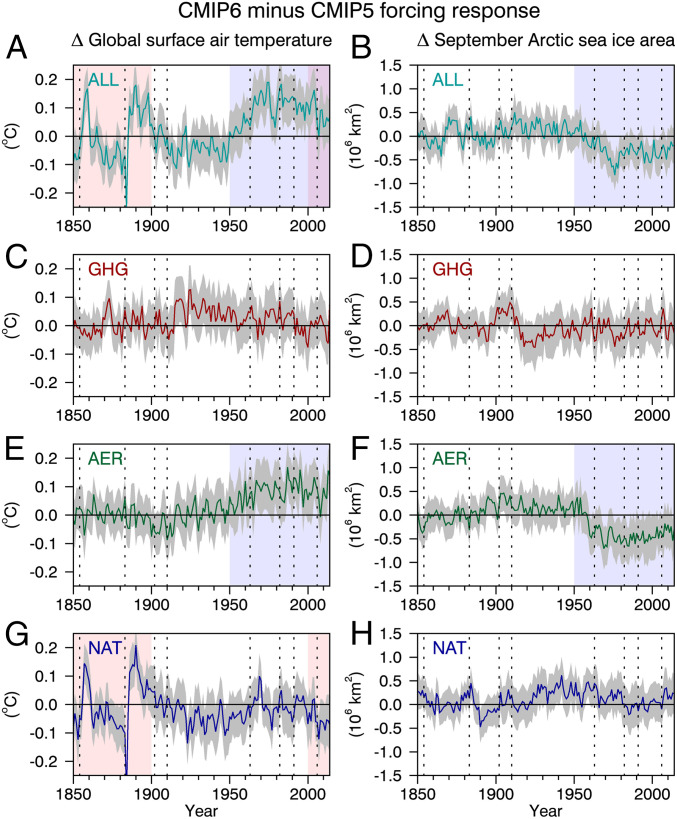
Differences in annual-mean and global-mean surface air temperature (*A*, *C*, *E*, and *G*) and September Arctic sea ice area (*B*, *D*, *F*, and *H*) between a 50-member ensemble of CanESM5 simulations employing CMIP6 forcings and a 10-member ensemble employing CMIP5 forcings. The solid colored curves are ensemble-mean differences and the gray shadings are 95% confidence intervals on the ensemble-mean differences. ALL, GHG, AER, and NAT denote simulations employing all forcings (*A* and *B*), greenhouse gases only (*C* and *D*), anthropogenic aerosols only (*E* and *F*), and volcanic and solar influences only (*G* and *H*), respectively. The vertical dotted lines are as in Fig. 1. The red shaded period is the period when significant differences exist that are attributable to volcanic forcing differences. The blue shaded period is when significant differences exist that are attributable to anthropogenic aerosol forcing differences.

We begin with GSAT behavior in two periods: 1850 to 1900 and 1950 to 2014 (the two shaded boxes in Fig. 3*A*). In the ALL experiments there are large ensemble-mean differences in GSAT between the CMIP5 and CMIP6 forced simulations over the last half of the 19th century (Fig. 3*A*). These differences are significant at the 95% confidence level and are due to differences in the natural forcings between CMIP5 and CMIP6 (Fig. 3*G*). Specifically, under CMIP6 forcings, 1) there is no evidence of a response to the 1854 Shiveluch eruption and 2) the initial cooling and subsequent warming response to the 1883 eruption of Krakatoa is larger and faster, respectively, than under CMIP5 forcing. We also mention that there is a marginally significant GSAT difference of −0.05±0.05 °C during the volcanically quiescent period from 1920 to 1960. This may reflect the influence of a difference in the background volcanic aerosol forcing between CMIP5 and CMIP6.

We now consider the GSAT differences over the last half of the 20th century and the early 21st century. Under CMIP6 forcing the surface is significantly warmer over most years from 1950 to 2014 than it is under CMIP5 forcing. Averaged over this period the ensemble-mean difference in the ALL simulations is 0.09±0.03 °C. The AER experiments reveal that this difference is due to a smaller cooling response to anthropogenic aerosols under CMIP6 forcing (Fig. 3*E*); the ensemble-mean difference in the AER simulations is 0.08±0.04 °C. To determine the specific aerosol loadings (e.g., sulfate aerosol, carbonaceous and/or organic aerosol) responsible for these differences would require additional simulations.

In the last decade of these simulations (i.e., 2005 to 2014) we note that there is a significant NAT difference of about 0.07±0.05 (Fig. 3*G*) due a sequence of small-to-moderate volcanic eruptions than are present under CMIP6 forcing but not under CMIP5 forcing. These small-to-moderate volcanoes received considerable attention in the context of the global warming slowdown (13–17). Our results are further evidence of an impact of these eruptions on global temperature.

Simulations with CMIP5 and CMIP6 forcings also show significant differences in Arctic sea ice area in September over the period from 1950 to 2014 (Fig. 3*B*). Averaged over this period, the ensemble-mean difference in Arctic sea ice area in the ALL simulations is about 0.27 ± 0.18 million ${km}^{2}$ smaller when CMIP6 forcings are applied than when CMIP5 forcings are. As in the case of GSAT, this is consistent with reduced cooling by anthropogenic aerosols in the CMIP6 simulations. This reduction in cooling leads to a decrease in Arctic sea ice area of 0.36 ± 0.26 million ${km}^{2}$ (Fig. 3*F*).

## Future Period (2015 to 2100)

For their future projections, models apply forcing scenarios that are developed and updated from one version of CMIP to the next. These are storylines of projected socioeconomic global changes up to 2100 (and beyond) that are turned into emission and/or concentration pathways for use in climate models. In CMIP5, these were referred to as representative concentration pathways (RCPs) (18), and in CMIP6 they are designated as shared socioeconomic pathways (SSPs) (1). Here we consider three pathways that are common, but not identical, between CMIP5 and CMIP6. These are RCP2.6, RCP4.5, and RCP8.5 from CMIP5 and SSP1-2.6, SSP2-4.5, and SSP5-8.5 from CMIP6. These RCP and SSP pairs of forcing scenarios are designed such that they approximately share the same long-term global average radiative forcing. For example, RCP2.6 and SSP1-2.6 should both have overall radiative forcing at the end of the 21st century equal to about 2.6 W$\cdot m^{-2}$.

The scenario simulations that we consider here are continuations of the ALL historical simulations discussed in the previous section. Under CMIP5 forcings we have 10 simulations and under CMIP6 forcings we have 50 simulations for each of the considered scenarios. Following the CMIP6 forcing protocol, each RCP and SSP simulation has a background volcanic aerosol component that was linearly “ramped” at the beginning of 2015 to the background volcanic aerosol value used in the respective preindustrial control simulation by 2024. We include the ramp in both sets of simulations here to isolate the effects of the RCP and SSP forcing differences.

**Fig. 4.**
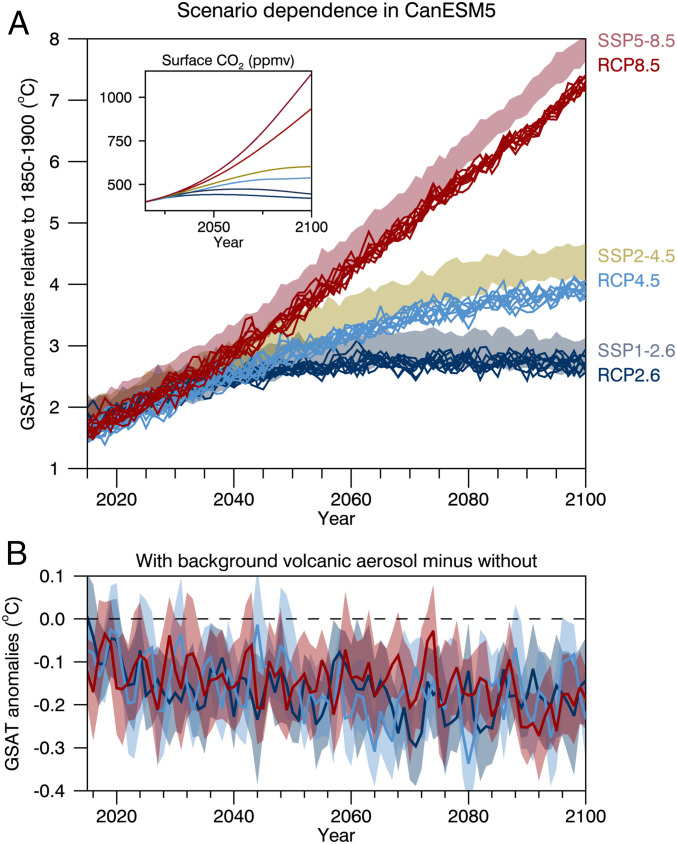
(*A* and *B*) Time series of anomalies in annual-mean and global-mean surface air temperature in a 50-member set of CanESM5 simulations employing SSP forcings and in a 10-member set employing RCP forcings (*A*) and differences between a 10-member set of CanESM5 simulations employing RCP forcings with and without background volcanic aerosols forcing (*B*). In *A* the colored shadings denote ranges across the simulations and colored lines indicate individual simulations. *Inset* shows the evolution of the prescribed annual-mean and global-mean carbon dioxide concentration near the surface. In *B* the solid colored curves are ensemble-mean differences and the gray shading is the 95% confidence interval on the ensemble-mean differences.

While the SSP and RCP “pairs” have equivalent radiative forcing in 2100, they have different end-of-century ${CO}_{2}$ concentrations (Fig. 4 *A*, *Inset*). At year 2100, the surface ${CO}_{2}$ concentration in SSP1-2.6, SSP2-4.5, and SSP5-8.5 is 25, 64, and 199 ppmv larger than in RCP2.6, RCP4.5, and RCP8.5, respectively. These ${CO}_{2}$ differences, and other GHG differences that may even be partly compensating, impact both the amount and time history of global warming (Fig. 4*A*). For example, averaged over the period from 2081 to 2100, the ensemble-mean warming in SSP1-2.6, SSP2-4.5, and SSP5-8.5 is 0.16 °C, 0.47 °C, and 0.55 °C greater than in RCP2.6, RCP4.5, and RCP8.5, respectively. These ensemble-mean differences are all highly statistically significant. These results are verification and quantification, using a comprehensive Earth System Model, of earlier results based on a simple climate model emulator (19).

As with our preindustrial control simulations, we now consider the role of background volcanic aerosols in future projections of GSAT. As mentioned above, the CMIP6 protocol for background volcanic aerosols was applied for both the SSP and RCP simulations to ensure a like-for-like comparison (Fig. 4*A*). The actual CMIP5 protocol did not include background volcanic aerosols in the RCP simulations. To assess the impact of this choice we initiated a second set of 10 simulations for each RCP that do not include a background volcanic aerosol contribution. Averaged over the period from 2081 to 2100, the ensemble-mean global temperature in the RCP2.6, RCP4.5, and RCP8.5 simulations with volcanic aerosols is 0.19 °C, 0.17 °C, and 0.20 °C cooler than in the RCP2.6, RCP4.5, and RCP8.5 simulations without volcanic aerosols, respectively (Fig. 4*B*). These ensemble-mean differences are also all highly statistically significant.

The implication of these volcanic forcing results is that if we were comparing SSP simulations against RCP simulations where the RCP simulations strictly adhered to the CMIP5 forcing protocol, then the percentage of differences in GSAT would have been about one-third smaller than shown in Fig. 4*A*. Specifically, the ensemble-mean warming averaged from 2081 to 2100 in SSP1-2.6 would be statistically indistinguishable from that in RCP2.6, while the warming in SSP2-4.5 and SSP5-8.5 would only be 6.9 and 5.1% greater than in RCP4.5 and RCP8.5, respectively. Finally, it is worthwhile pointing out that whereas CMIP5 had a repeating solar cycle in the future, CMIP6 uses a prediction of 21st century solar activity with multidecadal variations included (9). The effect of these multidecadal variations on global climate is likely to be small, but this would need to be verified with further targeted experiments.

## Forcing Uncertainty versus Model Uncertainty

We have found significant differences in the evolution of simulated global surface temperature and Arctic sea ice area between simulations performed with the same model using different applied forcings. This is true of preindustrial control, historical, and future simulations. To provide perspective on the magnitude of these differences, we compare the 10 CanESM5 historical simulations with a set of 5 CanESM2 (20) historical simulations. Both sets of simulations have identical CMIP5 ALL forcing, and both sets have no background volcanic aerosol forcing in their respective preindustrial control simulations. Any differences that are outside of internal variability are solely attributable to differences in the climate models rather than to differences in the prescribed forcings.

CanESM5, the current version of the Canadian Centre for Climate Modelling and Analysis (CCCma) global model, is a major update to the CanESM2 model that was used for CMIP5. The update includes improvements to the atmosphere, land surface, and terrestrial ecosystem models and the implementation of completely new models for the ocean, sea ice, and marine ecosystems (*Materials and Methods*). In CanESM5, the long-term temperature change for a doubling of atmospheric ${CO}_{2}$ concentration, or equilibrium climate sensitivity, is much higher than in CanESM2 (5.7 °C versus 3.7 °C) (4). Given the different model components and response properties, CanESM5 and CanESM2 are for all intents and purposes different models.

**Fig. 5.**
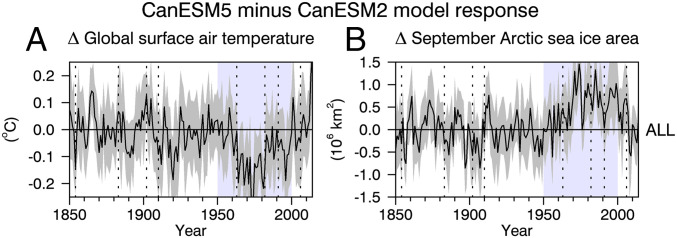
(*A* and *B*) Differences in annual-mean and global-mean surface air temperature (*A*) and September Arctic sea ice area (*B*) between a 10-member ensemble of CanESM5 simulations and a 5-member ensemble of CanESM2 simulations. Each simulation was run with the same CMIP5 forcings. The solid black curves are ensemble-mean differences and the gray shadings are 95% confidence intervals on the ensemble-mean differences. The vertical dotted lines are as in Fig. 1. The blue shaded period is the only extended time period when significant differences exist.

In terms of global temperature and Arctic sea ice, the only extended time period showing significant differences between CanESM2 and CanESM5 is over the second half of the 20th century (Fig. 5). During this period, CanESM2 is cooler on average than CanESM5 by about 0.08±0.04 °C and has about 0.52 ± 0.20 million ${km}^{2}$ more Arctic sea ice. These differences, obtained with two model versions, are about equal and opposite to the differences obtained using CanESM5 under CMIP5 and CMIP6 forcings. These results provide clear evidence that the uncertainty in global change arising between different forcing estimates can be as large as the uncertainty arising from different model versions.

## Implications

Our analysis has identified several significant climate implications of changes in the stratospheric and tropospheric aerosols specified in the latest-generation CMIP6 climate model simulations. These simulations will underpin much of the IPCC Sixth Assessment Report. Our comparison involves the CMIP5 simulations, which were evaluated in the IPCC Fifth Assessment Report (5, 21). The CMIP5 simulations did not include background volcanic aerosol (in most cases) in their control (or future) simulations. In contrast, CMIP6 simulations use background volcanic aerosol levels (the average volcanic aerosol loading from the historical integration) in their control and future simulations. While the choice of stratospheric aerosol level specified in the CMIP6 preindustrial control may seem academic, our analysis shows that it has substantial real-world implications. For example, this difference alone gives rise to 0.10±0.07 °C of additional warming in 2005 to 2014 compared to 1850 to 1900 in the CanESM5 CMIP6 simulations, since the model starts from a cooler state in 1850. Such a change would result in a ∼5% decrease in the cumulative carbon emissions budgets for 2 °C warming calculated using simulated warming relative to an 1861 to 1880 base period, as in the IPCC Fifth Assessment Report (3).

The choice of stratospheric aerosol level specified in the CMIP6 preindustrial control simulations also has a substantial effect on comparisons between global warming relative to 1850 to 1900 in CMIP5 models and observations presented in the IPCC Special Report on 1.5 °C (22). Additionally, this choice would influence attribution studies, which calculate scaling factors based on a comparison of simulated and observed temperature evolution since 1850 (23–25). If we are interested in making like-for-like comparisons of models and observations, then the most appropriate stratospheric aerosol background level would be what most closely approximates conditions over the previous several decades prior to the 1850 start of the historical simulation (26). Since we know there were several large volcanic eruptions during the first half of the 19th century (27), the higher level of aerosols specified in the CMIP6 control is probably more realistic, although careful analysis of paleo simulations extending prior to 1850 would be needed to identify whether even the CMIP6 control aerosol level is an underestimate. Ideally, historical simulations would be initiated from last millennium simulations with realistic time-varying volcanic forcing (28).

Since the CMIP6 models as a group tend to be cooler than observations and CMIP5 models in the second half of the 20th century (24, 29), it has been suggested that their average anthropogenic aerosol forcing in this period may be too strong (24, 29–31). In CanESM5, however, which was used to compare the responses to CMIP5 and CMIP6 aerosol forcings, the CMIP6 aerosol and aerosol precursor emissions result in a warmer climate from 1850 to 2000. This implies that if the aerosol forcing is responsible for the cooler temperatures on average in the CMIP6 models during this period, it is the stronger response to aerosols in the models which drives the cooling, rather than the emissions. This could be related to a number of different factors, including the aerosol cloud lifetime (second indirect) effect (4, 32) in a larger number of CMIP6 models (relative to CMIP5). Taken together, these results suggest that further investigation will be required to better understand aerosol forcing and response differences between CMIP5 and CMIP6. The ultimate goal is to derive stronger observationally based constraints on the patterns, size, and evolution of anthropogenic forcing and response (33).

Consistent with previous analyses (18, 19), we find that the SSP1-2.6, SSP2-4.5, and SSP5-8.5 scenarios result in substantially stronger warming than the RCP scenarios with nominally equivalent levels of 2100 radiative forcing. This is due to the higher levels of ${CO}_{2}$ in the SSPs. A countervailing effect is the increase in the background stratospheric aerosol loading assumed in the CMIP6 SSP simulations, which results in a substantial decrease in projected warming (of 0.19±0.04 °C) compared to CMIP5. Given observed global-mean warming to date relative to preindustrial of around 1.0 °C (22), our results imply that remaining carbon emissions budgets for a 1.5 °C temperature rise relative to preindustrial calculated from the CMIP6 simulations would be ∼20% larger than those in CMIP5. Similarly, given an anthropogenic warming trend of approximately 0.2 °C/decade (22, 25), calculated times of exceedance of 1.5 °C and 2.0 °C thresholds based on the CMIP6 simulations are expected to be up to about 10 y later than those calculated assuming CMIP5 scenarios. If we calculate and apply remaining carbon budgets from CMIP6 in this way, then we effectively are relying on future volcanic eruptions to help keep the global temperature increase to below the Paris thresholds.

Overall, our results demonstrate that differences in forcing and experimental design between CMIP5 and CMIP6 have a significant impact on the resulting climate simulations. These findings need to be carefully considered when interpreting results from the CMIP6 experiments and applying the results to guide climate policy. Going forward, it is critically important that coordinated multimodel efforts be undertaken to better quantify forcing uncertainties, as has been done in the past with regard to internal variability (34), volcanic and anthropogenic aerosol response (35, 36), and radiative forcing (37) uncertainties.

## Materials and Methods

We primarily use output from version 5 of the Canadian Earth System Model, denoted CanESM5 (4). This model was developed in the CCCma. In CanESM5, the atmosphere is represented by the Canadian Atmosphere Model (CanAM5), which incorporates the Canadian Land Surface Scheme (CLASS) and the Canadian Terrestrial Ecosystem Model (CTEM). The ocean is represented by a CCCma-customized version of the Nucleus for European Modeling of the Ocean (NEMO) model, with ocean biogeochemistry represented by the Canadian Model of Ocean Carbon (CMOC). The atmosphere and ocean components are coupled by the Canadian Coupler (CanCPL). The resolution of CanESM5 is about 2.8 ° in the atmosphere and about 1 ° in the ocean. We also use output from version 2 of the Canadian Earth System Model, denoted CanESM2 (20). CanESM2 has completely different models for the ocean, sea ice, and marine ecosystems than CanESM5 but has similar resolution. Note that the jump from version 2 to version 5 was made to reconcile internal model version labeling with the version label released to the public.

### Preindustrial Control Output.

To use CanESM5 with CMIP5 forcings, we consider the last 1,400 y of a 1,600-y-long preindustrial control simulation that includes no background volcanic aerosol forcing. From this 1,400-y preindustrial control segment we launched 10 simulations, each separated by 50 y. Each of these 10 runs includes a background volcanic aerosol contribution equal to the volcanic aerosol forcing averaged from 1850 to 2005 in the standard CMIP5 historical simulations. The 10 simulations were aligned in time and plotted in Fig. 2*A* (in black). To facilitate a like-for-like comparison, we compare this set of 10 simulations, each with background volcanic aerosol forcing, with 10 randomly selected 400-y-long segments from the 1,400-y-long simulation, each without background volcanic aerosol forcing. The 10 segments were also aligned in time and plotted in Fig. 2*A* (in blue).

### Historical Output.

We use CanESM5 to perform four 50-member sets of simulations from 1850 to 2014 with CMIP6 historical forcings. The fours sets are denoted ALL, GHG, AER, and NAT and they employ all major natural and anthropogenic forcings as well as greenhouse gas only, anthropogenic aerosols only, and natural only external factors (volcanic and solar), respectively. We also employ CanESM5 to perform four 10-member sets of simulations using CMIP5 historical forcings from 1850 to 2005 and RCP4.5 forcings from 2006 to 2014. Finally, we use CanESM2 and consider a 5-member set of simulations using CMIP5 all historical forcings from 1850 to 2005 and RCP4.5 forcings from 2006 to 2014.

### Future Output.

Using CanESM5 we generate three 50-member sets of simulation from 2015 to 2100 following SSP1-2.6, SSP2-4.5, and SSP5-8.5, as defined under CMIP6. Here, SSP*x–y* is such that *x* denotes a specific SSP as defined under CMIP6 (19) and *y* denotes the forcing pathway defined by its long-term global average radiative forcing level. Correspondingly, we use CanESM5 to perform three 10-member sets of simulations from 2006 to 2100 with RCP2.6, RCP4.5, and RCP8.5. Here, RCP*x* is such that the *x* denotes the long-term global average radiative forcing level (times 10) for a given RCP as defined under CMIP5 (18).

### Aerosol Scheme in CanESM5.

Concentrations of different types of natural and anthropogenic tropospheric aerosols are simulated in CanESM5, including sulfate, black and organic carbon, sea salt, and mineral dust (38). A bulk aerosol scheme is used, which accounts for emissions, transport, deposition, and gas-phase and aqueous-phase chemical reactions for sulfur. Concentrations of atmospheric dimethyl sulfide (DMS) in the ocean are specified, in addition to emissions from noneruptive volcanoes and wildfires. Sulfate and carbonaceous aerosols are internally mixed to simulate interactions with radiation, based on Maxwell Garnett theory. The first and second indirect effects of sulfate aerosols are simulated, using a semiempirical parameterization of cloud droplet number concentration.

### Observed Global Surface Temperature.

We use HadCRUT5.0, a new version of the Met Office Hadley Center and Climatic Research Unit global surface temperature dataset for 1850 to 2014 (39).

### Observed Arctic Sea Ice Area.

We use NSIDC3.0, the National Snow and Ice Data Center dataset of Arctic sea ice area for the period from 1979 to 2014.

## Data Availability

All datasets used here are publicly available. The HadCRUT5 dataset of observed global surface temperature are provided at the United Kingdom Meteorological Office (https://www.metoffice.gov.uk/hadobs/hadcrut5/). The NSIDC3.0 dataset of observed Arctic sea ice area are available at the National Snow & Ice data Center (https://nsidc.org/data/G02135/versions/3). The CanESM2 and CanESM5 global surface temperature and Arctic sea ice area were calculated from model simulations that are available from the Earth System Grid Federation (ESGF) (https://esgf-node.llnl.gov/projects/cmip5/ for CanESM2 and https://esgf-node.llnl.gov/projects/cmip6/ for CanESM5).
